# Agreement and disagreement in a non-classical world

**DOI:** 10.1098/rsta.2023.0004

**Published:** 2024-03-18

**Authors:** Adam Brandenburger, Patricia Contreras-Tejada, Pierfrancesco La Mura, Giannicola Scarpa, Kai Steverson

**Affiliations:** ^1^ Stern School of Business, Tandon School of Engineering, NYU Shanghai, New York University, New York, NY 10012, USA; ^2^ Instituto de Ciencias Matemáticas, 28049 Madrid, Spain; ^3^ HHL Leipzig Graduate School of Management, 04109 Leipzig, Germany; ^4^ Escuela Técnica Superior de Ingeniería de Sistemas Informáticos, Universidad Politécnica de Madrid, 28031 Madrid, Spain; ^5^ DCI Solutions, Aberdeen, MD 21005, USA

**Keywords:** Bayesian agents, common certainty, signed probabilities, quasi-probabilities, agreement and disagreement, communication

## Abstract

The Agreement Theorem Aumann (1976 *Ann. Stat.* **4**, 1236–1239. (doi:10.1214/aos/1176343654)) states that if two Bayesian agents start with a common prior, then they cannot have common knowledge that they hold different posterior probabilities of some underlying event of interest. In short, the two agents cannot ‘agree to disagree’. This result applies in the classical domain where classical probability theory applies. But in non-classical domains, such as the quantum world, classical probability theory does not apply. Inspired principally by their use in quantum mechanics, we employ signed probabilities to investigate the epistemics of the non-classical world. We find that here, too, it cannot be common knowledge that two agents assign different probabilities to an event of interest. However, in a non-classical domain, unlike the classical case, it can be common certainty that two agents assign different probabilities to an event of interest. Finally, in a non-classical domain, it cannot be common certainty that two agents assign different probabilities, if communication of their common certainty is possible—even if communication does not take place.

This article is part of the theme issue ‘Quantum contextuality, causality and freedom of choice’.

## 1. Introduction

In the domain of classical probability theory, Aumann [1] proved the fundamental result that Bayesian agents cannot agree to disagree. Two agents, Alice and Bob, begin with a common prior probability distribution on a state space. Next, they each receive different private information about the true state and form their conditional (posterior) probabilities $q_{A}$ and $q_{B}$ of an underlying event of interest. Then, if these two values $q_{A}$ and $q_{B}$ are common knowledge between Alice and Bob, they must be equal: $q_{A}=q_{B}$. By ‘common knowledge’ is meant that Alice knows Bob’s probability is $q_{B}$, Bob knows Alice’s probability is $q_{A}$, Alice knows Bob knows her probability is $q_{A}$, Bob knows Alice knows his probability is $q_{B}$, and so on indefinitely. This is the Agreement Theorem.

The role of common knowledge in this result is crucial. To conclude that $q_{A}=q_{B}$, it is not sufficient that Alice and Bob know each other’s probabilities. It is not even enough that they know these probabilities, and know they know them to some high finite order. Examples in which this condition allows $q_{A}\neq q_{B}$ are well known in the interactive epistemology literature [2,3]. The condition of common knowledge is tight.

The Agreement Theorem is considered a basic requirement in classical epistemics. It has been used to show that two risk-neutral agents, starting from a common prior, cannot agree to bet with each other [4], to prove ‘no-trade’ theorems for efficient markets [5] and to establish epistemic conditions for Nash equilibrium [2].

In this paper, we ask what is the status of the Agreement Theorem when classical probability theory does not apply. In the physical domain, the canonical case is quantum mechanics, where the fundamental Bell’s Theorem [6] says that no ‘local hidden-variable’ theory can model the results of all quantum experiments. However, it can be shown [7] that if signed probabilities on states are allowed, then there is a phase-space representation for all no-signalling models [8], which is a family of models that (strictly) includes those realizable within quantum mechanics. A phase-space model can be thought of as a canonical hidden-variable model, where the different states are precisely the different values the hidden variable can take. A signed probability measure is a measure that can assign negative values to certain events, while still assigning probability 1 to the whole space.

In fact, the use of signed probabilities in quantum mechanics goes back even earlier, to the Wigner ‘quasi-probability distribution’ [9], which is widely used in quantum mechanics—for example, in the field of quantum optics [10]. Dirac [11] and Feynman [12] also promoted the use of quasi- or signed probabilities in quantum mechanical calculations. Kaszlikowski & Kurzyński [13] continue this tradition with their proposal for treating the ‘nebit’, which is a negative bit, as the basic unit of stochastic negativity.

Bell’s Theorem applies to a two-qubit system. Signed probabilities also arise in phase-space representations of a one-qubit system, under certain conditions. Brandenburger *et al.* [14] derive the qubit from an entropic uncertainty principle stated on phase space. In their framework, there are quantum states whose only phase-space representations that respect the uncertainty bound involve negative probabilities. Onggadinata *et al.* [15] derive the qubit with its full dynamics via an entropic invariance principle involving signed probabilities.

In probability theory, there is a finite analogue to the de Finetti representation theorem for infinite sequences of exchangeable random variables [16], if mixing is via a signed probability measure [17, pp. 46–47], [18–20]. Turning again to physics, this permits an exchangeability derivation of Fermi–Dirac statistics, paralleling an infinite exchangeability derivation of Bose–Einstein statistics [20,21].

Decision theory is another area in which signed probabilities have emerged. Perea [22] axiomatizes expected utility theory for conditional preference relations, where such a relation assigns to every possible probability measure on a (finite) set of states that the decision maker might hold, a preference relation over the decision-maker’s (finite) set of actions. The motivation is game theory, where one typically specifies the game matrix, and hence the players’ payoff or utility functions, but one thinks of a player as contemplating different beliefs (probability measures) they might hold concerning the actions chosen by other players. The question is when is such a conditional preference relation representable by a single utility function, with the expectation of utility taken under the given probability measure. Perea [22] proposes a set of axioms that yields such a representation, but the axiomatization requires the decision maker to entertain signed as well as ordinary probability measures on the states.

Ke & Zhao [23] include new representation results that may be applied to decision making under ambiguity. In the set-up with ambiguity, their ‘cautiously optimistic linear utility’ representation (equivalent to a utility function that is locally exactly linear almost everywhere) features a collection of sets of possibly signed (subjective) measures over states.

A crucial common feature in the quantum mechanical and statistical mechanical applications of signed probabilities is that all observable events must receive probability between 0 and 1. It is less clear-cut in the decision-theoretic application that this condition must hold, since the setting might be a one-off decision and probabilities could be subjective rather than frequentist. This said, in the subjective case, too, it would be decidedly unorthodox to allow negative probabilities on observable events. To be ‘conservative’, we shall require all observable events in the formalism of this paper to receive probability in [0,1].

Returning to the classical Agreement Theorem as our starting point, we establish three results, where the second and third use the ‘common certainty’ modality (to be defined later) in place of common knowledge:

(i) In a non-classical domain, with signed probabilities, and as in the classical domain, it cannot be common knowledge that two agents assign different probabilities to an event of interest.
(ii) In a non-classical domain, and unlike the classical domain, it can be common certainty that two agents assign different probabilities to an event of interest.
(iii) In a non-classical domain, it cannot be common certainty that two agents assign different probabilities to an event of interest, if communication of their common certainty is possible—even if communication does not take place.

We formulate and prove these results in the following sections of the paper.

## 2. Example

**Figure 1.**
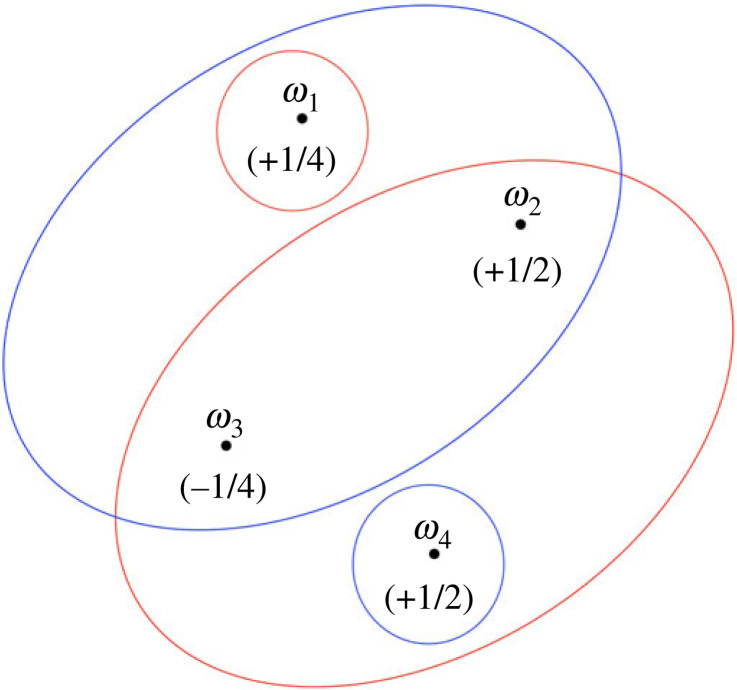
Singular disagreement in the non-classical world.

Figure 1 depicts an epistemic state space that contains a non-classical component. There are four states, labelled $\omega_{1}$ through $\omega_{4}$. There is a common prior, and the prior probabilities of the states are given in parentheses. Notice that the (prior) probability of state $\omega_{3}$ is negative, which cannot, of course, happen in a classical setting. There are two agents, Alice and Bob. Alice receives private information about the true state as represented by the red sets partitioning the state space, while Bob receives private information as represented by the blue sets. Finally, we are interested in the agents’ respective (conditional) probabilities of the event $E=\{\omega_{1},\omega_{3},\omega_{4}\}$ when the true state of the world is $\omega_{1}$.

Let p denote the common prior (which is a signed probability measure). When the true state is $\omega_{1}$, Alice’s information is $\left\{\omega_{1}\right\}$, so that her conditional probability of E is equal to

$$\frac{p(\{\omega_{1},\omega_{3},\omega_{4}\}\cap \{\omega_{1}\})}{p(\{\omega_{1}\})}=\frac{p(\{\omega_{1}\})}{p(\{\omega_{1}\})}=\frac{+(1/4)}{+(1/4)}=1.$$

Bob’s information is $\left\{\omega_{1},\omega_{2},\omega_{3}\right\}$, so that his conditional probability of E is equal to

$$\frac{p(\{\omega_{1},\omega_{3},\omega_{4}\}\cap \{\omega_{1},\omega_{2},\omega_{3}\})}{p(\{\omega_{1},\omega_{2},\omega_{3}\})}=\frac{p(\{\omega_{1},\omega_{3}\})}{p(\{\omega_{1},\omega_{2},\omega_{3}\})}=\frac{+(1/4)-(1/4)}{+(1/4)+(1/2)-(1/4)}=\frac{0}{+(1/2)}=0.$$

Thus, at state $\omega_{1}$, Alice assigns probability 1 to E and Bob assigns probability 0 to E. Next, we find the event, which we depict F, that Bob assigns probability 0 to E. We know that $\omega_{1}\in F$. Bob’s probability of E is again 0 at states $\omega_{2}$ or $\omega_{3}$. At state $\omega_{4}$, Bob’s probability of E is

$$\frac{p(\{\omega_{1},\omega_{3},\omega_{4}\}\cap \{\omega_{4}\})}{p(\{\omega_{4}\})}=\frac{p(\{\omega_{4}\})}{p(\{\omega_{4}\})}=\frac{+(1/2)}{+(1/2)}=1,$$

so that $F=\{\omega_{1},\omega_{2},\omega_{3}\}$. At state $\omega_{1}$, Alice’s probability of F is

$$\frac{p(\{\omega_{1},\omega_{2},\omega_{3}\}\cap \{\omega_{1}\})}{p(\{\omega_{1}\})}=\frac{p(\{\omega_{1}\})}{p(\{\omega_{1}\})}=\frac{+(1/4)}{+(1/4)}=1.$$

Thus, at state $\omega_{1}$, Alice assigns probability 1 to E while at the same time she assigns probability 1 to Bob’s assigning probability 0 to E. Say Alice is **certain of** an event E at a state $\omega_{i}$ if she assigns probability 1 to E, conditional on the information she has at state $\omega_{i}$. Then the scenario we have just constructed is one where there is a state at which Alice is certain of an event E, and Alice is certain Bob is certain of the complementary event $E^{c}$. Call this a situation of **singular disagreement** (like calling two probability measures mutually singular). Evidently, this phenomenon can arise in a non-classical environment. In the next section, we will verify that singular disagreement cannot arise in a (finite) classical environment.

In a model with negative probabilities, events that receive probability in [0,1] are observable in the sense that they can be associated to frequencies. In the example, all partition cells, namely $\left\{\omega_{1}\},\{\omega_{2},\omega_{3},\omega_{4}\},\{\omega_{1},\omega_{2},\omega_{3}\right\}$ and $\left\{\omega_{4}\right\}$, receive probability in (0,1]. So, they are observable and, in fact, strict positivity of these events ensures that the agents can also condition on them. The event E receives probability in (0,1], which means it is observable and non-trivial. We will consider observability further in §4.

## 3. General formulation

For the general case, let the state space be a finite set Ω, and let Alice and Bob have partitions of Ω denoted by $P_{A}$ and $P_{B}$, respectively. For ω∈Ω, the event $P_{A}(\omega )$ consists of the member of Alice’s partition that contains ω; similarly for $P_{B}(\omega )$. Let p denote the common (possibly signed) prior probability measure on Ω. We will assume throughout that all members of the partitions $P_{A}$ and $P_{B}$ receive non-zero probability, so that conditioning is well defined.

We begin with a remark about the classical domain.

**Remark 3.1.**

Suppose that p is non-negative and fix an event E. Let F be the event that Bob assigns probability 0 to E, i.e.

$$F=\{\omega {\;}^{\prime }\in \Omega :p(E|P_{B}(\omega {\;}^{\prime }))=0\}.$$

Then there is no state ω at which Alice assigns probability 1 to E∩F.

**Proof.**

Suppose there is such a state ω. Then $p(E|P_{A}(\omega ))=1$ and $p(F|P_{A}(\omega ))=1$. Note that we can write $F=\underset{i\in I}{⋃}\pi_{i}$ where each $\pi_{i}\in P_{B}$ and I is a (finite) index set. In particular, there is a $\pi_{i}\in P_{B}$ such that $p(E|\pi_{i})=0$ and $p(\pi_{i}|P_{A}(\omega ))>0$.

We now have three events A ($=P_{A}(\omega )$), B (=E) and C ($=\pi_{i}$) such that p(B∣A)=1, p(B∣C)=0 and p(C∣A)>0. From p(B∣A)=1, we get p(A∩(C∖B))=0. From p(B∣C)=0, we get p(A∩(B∩C))=0. It follows that p(A∩C)=0, contradicting p(C∣A)>0.

Remark 3.1 says that singular disagreement is impossible in the (finite) classical domain, verifying that this phenomenon is non-classical. The example of the previous section makes use of the fact that signed probability measures do not satisfy monotonicity. Specifically, in the proof just given, the step p(A∩(B∩C))=0 because p(B∣C)=0 fails with signed probabilities.

Next, we provide formal definitions of knowledge, common knowledge, certainty and common certainty.

**Definition 3.2.**

Alice **knows** an event E at state ω if $P_{A}(\omega )\subseteq E$.

At state ω, Alice’s information is that the true state lies in $P_{A}(\omega )$. It follows that the true state therefore lies in any superset of $P_{A}(\omega )$, i.e. that Alice knows all such events obtain. This is the standard definition of knowledge in the interactive epistemology literature. Some notation: the meet (finest common coarsening) of Alice’s and Bob’s partitions is written $P_{A}\wedge P_{B}$. The member of the meet that contains state ω is written $\left(P_{A}\wedge P_{B})(\omega \right)$.

**Definition 3.3.**

An event E is **common knowledge** between Alice and Bob at a state ω if $(P_{A}\wedge P_{B})(\omega )\subseteq E$.

This definition of common knowledge is easily shown to be equivalent to the recursive definition (Alice knows E occurs, Bob knows E occurs, Alice knows Bob knows E occurs, etc.). Aumann [1] proves this fact.

**Definition 3.4.**

Alice is **certain of** an event E at state ω if $p(E|P_{A}(\omega ))=1$.

At state ω, Alice’s information is that the true state lies in $P_{A}(\omega )$. She is certain of E if she assigns probability 1 to E, conditional on this information. This is the standard epistemic definition of certainty.

Next, fix an event E and probabilities $q_{A}$ and $q_{B}$. We define the event that it is common certainty that Alice assigns probability $q_{A}$ to E and Bob assigns probability $q_{B}$ to E. To do so, let

$$A_{0}=\{\omega \in \Omega :p(E|P_{A}(\omega ))=q_{A}\}$$

and

$$B_{0}=\{\omega \in \Omega :p(E|P_{B}(\omega ))=q_{B}\},$$

and, in addition, let

$$A_{n+1}=A_{n}\cap \{\omega \in \Omega :p(B_{n}|P_{A}(\omega ))=1\}$$

and

$$B_{n+1}=B_{n}\cap \{\omega \in \Omega :p(A_{n}|P_{B}(\omega ))=1\},$$

for n≥0. The set $A_{0}$ contains all the states where Alice assigns probability $q_{A}$ to E. The set $A_{1}$ contains all the states where the previous statement for Alice is true and, in addition, Alice is certain ‘Bob assigns probability $q_{B}$ to E’. The set $A_{2}$ contains all the states where the previous statement for Alice is true and, in addition, Alice is certain ‘Bob assigns probability $q_{B}$ to E and he is certain she assigns probability $q_{A}$ to E’, and so on. In this way, the set $A_{n}$ contains all the states where Alice has nth-order certainty. Likewise for Bob and the sets $B_{n}$, for all n.

**Definition 3.5.**

It is **common certainty** at a state $\omega^{*}$ that Alice assigns probability $q_{A}$ to E and Bob assigns probability $q_{B}$ to E if

$$\omega^{*}\in \overset{\infty }{\underset{n=0}{⋂}}A_{n}\cap \overset{\infty }{\underset{n=0}{⋂}}B_{n}.$$

A special case is where $q_{A}=q_{B}=1$. Then we can simply say that the event E is **common certainty** between Alice and Bob at $\omega^{*}$.

**Figure 2.**
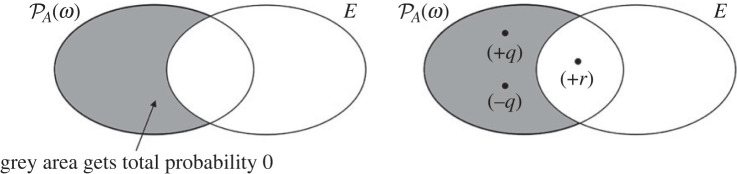
Classical and non-classical knowledge-certainty distinction.

It is clear that if Alice knows an event E at state ω, then she is certain of E at ω. It is also true that common knowledge of E implies common certainty of E. (Proof: we just gave the first step. Next, if Alice knows Bob knows E, then she knows Bob is certain of E, since knowledge is monotonic. From this, Alice is certain Bob is certain of E. The argument can be continued to all higher levels.) But certainty is a strictly weaker modality than knowledge. (Also, common certainty is strictly weaker than common knowledge, as we will see in theorem 4.2.) Figure 2 demonstrates this claim in two different instances—the first classical and the second non-classical. In both instances, Alice is certain of E but she does not know E.

## 4. Agreement and disagreement

We can now state and prove a knowledge-based Agreement Theorem for both the classical and non-classical domains.

**Theorem 4.1.**

*Fix a common prior (which may be a signed probability measure) and an event* E. *Suppose at a state* $\omega^{*}$ *it is common knowledge that Alice’s probability of* E *is* $q_{A}$ *and Bob’s probability of* E *is* $q_{B}$. *Then* $q_{A}=q_{B}$.

**Proof.**

The hypothesis of the theorem is that

$$(P_{A}\wedge P_{B})(\omega^{*})\subseteq A_{0}\cap B_{0}.$$

Now, we can write $(P_{A}\wedge P_{B})(\omega^{*})=\underset{i\in I}{⋃}\pi_{i}$ where each $\pi_{i}\in P_{A}$ and I is a (finite) index set. Since $(P_{A}\wedge P_{B})(\omega^{*})\subseteq A_{0}$, we have $p(E|\pi_{i})=q_{A}$ for all i∈I. We also have

$$p(E|(P_{A}\wedge P_{B})(\omega^{*}))=\sum_{j\in I}p(\pi_{j}|\underset{i\in I}{⋃}\pi_{i})\times p(E|\pi_{j}),$$

so that $p(E|(P_{A}\wedge P_{B})(\omega^{*}))$ is an affine combination of $q_{A}$’s and is therefore equal to $q_{A}$. We can run exactly the same argument with B in place of A to conclude that $p(E|(P_{A}\wedge P_{B})(\omega^{*}))=q_{B}$. It follows that $q_{A}=q_{B}$.

It is of note that we did not need to impose any observability conditions in this theorem, making it fully general. (If we did add the condition that members of $P_{A}$ and $P_{B}$ receive strictly positive—as opposed to non-zero—probability, then the affine combination in the proof would become a convex combination and the proof would be exactly that in Aumann [1].)

Theorem 4.1 is similar to results in Leifer & Duarte [24] on the impossibility of common knowledge of disagreement, established in the setting of generalized probability theory or GPT [25]. GPT is a multi-purpose operational framework for describing physical theories, including quantum mechanics.

In the classical domain, with a non-negative prior, there is also an Agreement Theorem for the certainty modality: if two agents have common certainty of each other’s probabilities of E, then these probabilities must be equal, just as with common knowledge. We do not give a direct proof here, since the result will be a corollary to our theorem 5.4 later. Taken together, theorem 4.1 (for the classical case of a non-negative prior) and the analog for common certainty indicate that the distinction between the knowledge and certainty modalities is ‘small’—at least, for current purposes—in the classical domain. But the distinction is very significant in the non-classical domain, because the Agreement Theorem for certainty no longer holds there, as we are about to see.

We suggest that the certainty modality in epistemics is at least as interesting as the knowledge modality. Certainty is subjective in that an agent can be certain of an event E, but E need not happen. Knowledge is objective and satisfies the truth axiom: if an agent knows E, then E must occur. The subjective modality seems more in line with the idea that Alice and Bob are Bayesian agents forming their personalistic beliefs, beliefs about beliefs, and so on, about some event. For Alice to know Bob’s beliefs (or knowledge) requires that she have direct information about his epistemic state. This introduces an ex post element to the analysis in the sense that Bob’s epistemic state would need to be observed by Alice (via some information flow). The certainty modality allows an ex ante analysis where agents form prospective beliefs about events, just as in Bayesian decision theory [26]. In any case, we think the point is made that the certainty modality is important to study and, as we now show, it is very different from the knowledge modality in the non-classical world.

**Theorem 4.2.**

*There is a structure* $\left(\Omega ,p,P_{A},P_{B}\right)$, *where* p *is a signed prior, and there is an event* E *and a state* ω *such that it is common certainty at* ω *that Alice and Bob hold different probabilities of* E.

**Proof.**

**Figure 3.**
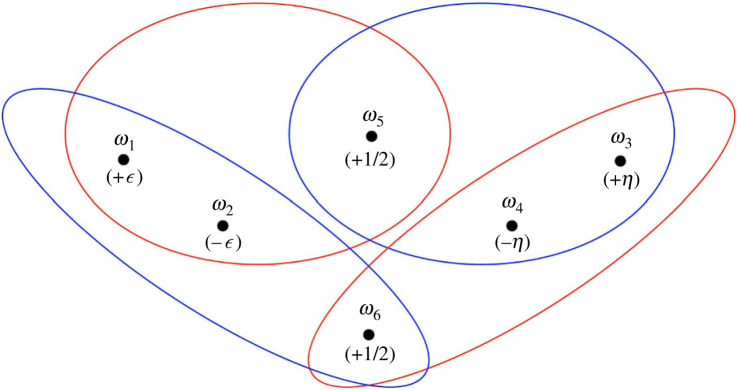
Common certainty of disagreement in a non-classical world.

The state space and prior are depicted in figure 3. As in our earlier example, Alice’s partition comprises the red sets and Bob’s partition comprises the blue sets. The event $E=\{\omega_{2},\omega_{4},\omega_{5},\omega_{6}\}$ and the true state is $\omega_{5}$. The numbers ϵ and η are small and positive with ϵ≠η. Set

$$A_{0}=\{\omega \in \Omega :p(E|P_{A}(\omega ))=1-2\epsilon \}=\{\omega_{1},\omega_{2},\omega_{5}\}$$

and

$$B_{0}=\{\omega \in \Omega :p(E|P_{B}(\omega ))=1-2\eta \}=\{\omega_{3},\omega_{4},\omega_{5}\},$$

so that

$$A_{1}=A_{0}\cap \{\omega \in \Omega :p(B_{0}|P_{A}(\omega ))=1\}=\{\omega_{1},\omega_{2},\omega_{5}\}$$

and

$$B_{1}=B_{0}\cap \{\omega \in \Omega :p(A_{0}|P_{B}(\omega ))=1\}=\{\omega_{3},\omega_{4},\omega_{5}\},$$

from which $A_{n+1}=A_{n}$ and $B_{n+1}=B_{n}$ for all n≥1. It follows that $\omega_{5}\in \overset{\infty }{\underset{n=0}{⋂}}A_{n}\cap \overset{\infty }{\underset{n=0}{⋂}}B_{n}$. At state $\omega_{5},$ it is common certainty between Alice and Bob that she assigns probability 1−2ϵ to E while he assigns probability 1−2η to E, which proves the theorem.\qedhere

Note that, by theorem 4.1, the agents’ probabilities of E cannot be common knowledge at $\omega_{5}$ (because then the probabilities must be the same). Alternatively, this can be checked directly via the definition of common knowledge in terms of the join of $P_{A}\wedge P_{B}$ (which is the whole space). So, this example also serves to establish the claim that common certainty is strictly weaker than common knowledge. Note also that the example exhibits a high degree of observability: all members of $P_{A}$ and $P_{B}$ get strictly positive probability (+1/2). The event of interest E gets probability 1−ϵ−η>0.

## 5. Communication

Common knowledge and common certainty are different from communication between agents. If Alice announces the probability she assigns to an event of interest E, then this communicates information to Bob and he can update his partition $P_{B}$ to incorporate this information. Vice versa if Bob communicates to Alice, who can then announce new probabilities. This process could continue. The communication of probabilities this way was first studied by Geanakoplos & Polemarchakis [3].

Let us try to go down this road in the non-classical environment of this paper. Go back to the structure in figure 3. The event of interest is $E=\{\omega_{2},\omega_{4},\omega_{5},\omega_{6}\}$ as before, but now the true state is $\omega_{1}$. Alice begins by announcing her probability 1−2ϵ of E to Bob. When Bob hears this announcement, he tries to make an inference about what information Alice has—specifically, whether she has the information $\left\{\omega_{1},\omega_{2},\omega_{5}\right\}$ (as, in fact, she does) or the information $\left\{\omega_{3},\omega_{4},\omega_{6}\right\}$ (which she does not), or whether he cannot tell which piece of information she has. Start with the second case. Bob can reason that, in this case, Alice would have announced a probability 1−2η of E. Since she did not, he can infer that she did not observe $\left\{\omega_{3},\omega_{4},\omega_{6}\right\}$. Next is the case that Alice has the information $\left\{\omega_{1},\omega_{2},\omega_{5}\right\}$. Bob can reason that, in this case, Alice would have announced a probability 1−2ϵ of E. Since she did just this, he can infer that she indeed observed $\left\{\omega_{1},\omega_{2},\omega_{5}\right\}$. Summing up, Bob will update his own information to

$$\{\omega_{1},\omega_{2},\omega_{6}\}\cap \{\omega_{1},\omega_{2},\omega_{5}\}=\{\omega_{1},\omega_{2}\}.$$

Now ask what probability of E Bob will announce. We can try to calculate this as

$$\frac{p(E\cap \{\omega_{1},\omega_{2}\})}{p(\{\omega_{1},\omega_{2}\})}=\frac{-\epsilon }{0},$$

which is obviously ill defined.

What has gone ‘wrong’ in this process is that Bob is unable to process the information contained in Alice’s announcement in a meaningful way. There is the negative number −ϵ in the numerator and the 0 in the denominator. We argue that both features pose a conceptual problem. In systems where the agents are able to communicate about an event of interest, we propose that those communications should lead to well-defined and classical conditional probabilities regarding that event. That is, the resulting conditional probabilities should all lie in the interval [0,1]. Communication—even if it concerns a non-classical system—should be considered observable and therefore classical.

We next impose a condition on our epistemic structures that ensures all communication regarding an event of interest is classical. In doing this, we depart from the Geanakoplos & Polemarchakis [3] protocol in a key way. We focus on the initial announcements that Alice and Bob can make, i.e. announcements relative to their initial partitions $P_{A}$ and $P_{B}$, respectively. But we allow that these announcements might be of any order. That is, Alice might announce her probability of E, or she might announce her certainty (or not) of what Bob’s probability of E is, or she might announce her certainty (or not) about Bob’s certainty, and so on. Likewise for Bob. This is different from the Geanakoplos–Polemarchakis protocol, which permits announcements only of (conditional) probabilities of E, but allows these announcements to continue, as Alice and Bob successively update their information. With our approach, we are able to (re-)establish an agreement theorem even in the non-classical domain. We leave it as open whether or not such a result is possible if an appropriate notion of classicality is imposed on the Geanakoplos–Polemarchakis protocol adapted to our setting.

Formally, for all n≥0, let

$$M_{A}^{\left(n\right)}=\{A_{n},A_{n}^{c}\}$$

and

$$M_{B}^{\left(n\right)}=\{B_{n},B_{n}^{c}\},$$

where $A_{n}$ and $B_{n}$ are the sets defined back in §3.

**Definition 5.1.**

For any π,E⊆Ω, say π is **regular with respect to** E if p(π)≥0 and p(π∩E) lies in [0,p(π)].

**Definition 5.2.**

A structure $\left(\Omega ,p,P_{A},P_{B}\right)$ is **communication-enabled with respect to** E if, for each n≥0, all $\pi \in P_{A}\vee M_{B}^{\left(n\right)}$ and all $\pi^{\prime }\in P_{B}\vee M_{A}^{\left(n\right)}$ are regular with respect to E.

These definitions capture our requirement that if an agent were to communicate their initial certainty at any level, the calculation that the other agent would then make is classical.

**Remark 5.3.**

Fix $\pi ,\pi^{\prime }\subseteq \Omega$ with $\pi \cap \pi^{\prime }=\emptyset$. Then if π and $\pi^{\prime }$ are both regular with respect to E, so is $\pi \cup \pi^{\prime }$.

We have assumed throughout that all members of $P_{A}$ and $P_{B}$ receive non-zero probability. Definition 5.2 assumes non-negative probabilities. So, at this point, we are assuming that all members of $P_{A}$ and $P_{B}$ receive strictly positive probability. This implies that the agents are able to observe and condition on their own information using the rules of ordinary probability.

**Theorem 5.4.**

*Fix a structure* $\left(\Omega ,p,P_{A},P_{B}\right)$ *that is communication-enabled with respect to* E *and suppose that at a state* $\omega^{*}$ *it is common certainty that Alice’s probability of* E *is* $q_{A}$ *and Bob’s probability of* E *is* $q_{B}$. *Then* $q_{A}=q_{B}$.

**Proof.**

Begin by defining $A_{n}$ and $B_{n}$, for n≥0, as before. Since Ω is finite, there is an N (finite) such that for all n≥N, $A_{n+1}=A_{n}$ and $B_{n+1}=B_{n}$. We have

$$A_{N+1}=A_{N}\cap \{\omega \in \Omega :p(B_{N}|P_{A}(\omega ))=1\}=A_{N},$$

from which $p(B_{N}|P_{A}(\omega ))=1$ for all $\omega \in A_{N}$.

Now $A_{N}=\underset{i\in I}{⋃}\pi_{i}$ where each $\pi_{i}\in P_{A}$ and I is a (finite) index set. We just saw that $p(B_{N}|\pi_{i})=1$ for all such $\pi_{i}$. But $p(B_{N}|A_{N})$ is a convex combination of the $p(B_{N}|\pi_{i})$’s, so $p(B_{N}|A_{N})=1$. It follows that $p(A_{N}\setminus B_{N})=0$, which we will use shortly.

Observe that $A_{N}\subseteq A_{0}$ and so $p(E|\pi_{i})=q_{A}$ for these same $\pi_{i}$. By a second convex combination argument, $p(E|A_{N})=q_{A}$.

Next, observe that $\left\{A_{N},A_{N}^{c}\right\}$ is a coarsening of $P_{A}$ (by definition of the $A_{n}$’s). From this and $M_{B}^{\left(N\right)}=\{B_{N},B_{N}^{c}\}$, it follows that $\left\{A_{N}\setminus B_{N},{\left(A_{N}\setminus B_{N}\right)}^{c}\right\}$ is a coarsening of $P_{A}\vee M_{B}^{\left(N\right)}$. By the hypothesis of the theorem and remark 5.3, it follows that $A_{N}\setminus B_{N}$ is regular with respect to E. Using $p(A_{N}\setminus B_{N})=0$, it follows that $p((A_{N}\setminus B_{N})\cap E)=0$, and so $p(E\cap A_{N}\cap B_{N})=p(E\cap A_{N})$. Again using $p(A_{N}\setminus B_{N})=0$, we get $p(A_{N}\cap B_{N})=p(A_{N})>0$ (the set $A_{N}$ is a union of members of $P_{A}$). We conclude that $p(E|A_{N}\cap B_{N})=p(E|A_{N})=q_{A}$. We can run exactly the same argument with B in place of A to conclude that $p(E|A_{N}\cap B_{N})=q_{B}$. It follows that $q_{A}=q_{B}$.

We are not committed to theorem 5.4 over theorem 4.2. There is no formal or even obvious conceptual inconsistency in the set-up of theorem 4.2. Still, it is interesting to discover from theorem 5.4 that if we impose the requirement that each agent be able to process classically an announcement by the other agent of their certainty at any level, then the non-classical phenomenon of common certainty of disagreement disappears. There is a subtle point here. No actual communication needs to take place. Rather, we can think of our requirement as saying that it would be possible for the two agents to confirm their disagreement, not just have common certainty of their disagreement, if they wanted to.

A corollary to theorem 5.4 is that common certainty of disagreement is impossible in the classical world, as we mentioned earlier. This follows because the condition of being communication-enabled is automatically satisfied in the case of non-negative probabilities.

Consider another communication scenario: there is a third agent, Charlie, who starts out with no information about the true state. Alice and Bob are able to communicate with Charlie, but not with each other. (They do not necessarily undertake the communication.) We can ask if this scenario, too, rules out common certainty of disagreement. Here is the appropriate analogue to definition 5.2.

**Definition 5.5.**

A structure $\left(\Omega ,p,P_{A},P_{B}\right)$ is **third-party communication-enabled with respect to** E if, for each n≥1, each $\pi \in M_{A}^{\left(n\right)}\vee M_{B}^{\left(n\right)}$ is regular with respect to E.

The idea is that the third party, Charlie, starts with the trivial partition {Ω,∅} and is then able to make classical calculations with the information which announcements by Alice and Bob might give him. Alice and Bob do not communicate with each other.

**Theorem 5.6.**

*Fix a structure* $\left(\Omega ,p,P_{A},P_{B}\right)$ *that is third-party communication-enabled with respect to* E *and suppose that at a state* $\omega^{*}$ *it is common certainty that Alice’s probability of* E *is* $q_{A}$ *and Bob’s probability of* E *is* $q_{B}$. *Then* $q_{A}=q_{B}$.

**Proof.**

From $M_{A}^{\left(N\right)}=\{A_{N},A_{N}^{c}\}$ and $M_{B}^{\left(N\right)}=\{B_{N},B_{N}^{c}\}$ it follows that $\left\{A_{N}\setminus B_{N},{\left(A_{N}\setminus B_{N}\right)}^{c}\right\}$ is a coarsening of $M_{A}^{\left(N\right)}\vee M_{B}^{\left(N\right)}$. Using the hypothesis of the theorem and remark 5.3, we conclude that $A_{N}\setminus B_{N}$ is regular with respect to E. The rest of the proof follows exactly the proof of theorem 5.4.

Finally, in this section, we note that a complete treatment of updating of probabilities in the non-classical domain would require the development of some new probability theory. The issue is that, as can happen in a classical setting, an agent may come to learn that an event to which they had assigned probability 0 actually obtains. We saw this in the example above where we got a zero in the denominator of Bob’s updated probability. In the classical domain, the answer to the probability-0 problem is to move to the concept of a conditional probability system [27]. This is a family of probability measures—one measure for each event an agent might learn, including events to which the agent assigns probability 0. What would be needed in our non-classical domain is an extension of the concept of a conditional probability system to signed probabilities, which would be an exercise in pure probability theory that, to the best of our knowledge, has not been undertaken.

## 6. Conclusion

We end with some comments on the realizability of common certainty of disagreement (CCD) as in theorem 4.2. In the physical domain, it can be shown that CCD is impossible when observing quantum systems, but possible when observing superquantum (no-signalling) systems [28]. In the language of this paper, we can say that quantum mechanics somehow controls the ‘extent’ of negativity in phase-space probability representations so that CCD cannot arise. This finding suggests there may be promise in proposing the impossibility of CCD (of ‘agreeing to disagree’) as an axiom in the program to derive quantum mechanics from underlying physical principles. (See [28] for further discussion and references to the axiomatization programme.)

It would be interesting to connect this paper to the study of contextuality scenarios in quantum and superquantum systems. In particular, Cabello [29] extends the usual contextuality scenario involving a single observer to allow for a copy of the system with a second observer. He identifies a principle he calls ‘global exclusivity’ that exactly identifies the maximum quantum violation of certain non-contextuality inequalities. Combining formal epistemics as in the current paper with this physical principle could be a promising direction.

In the setting of decision theory—more precisely, multi-person decision theory—theorem 4.2 indicates that if we equip agents with signed probability measures, we can get highly non-classical behaviour, such as betting between risk-neutral agents. With this in mind, we wonder whether it might be interesting to elevate the impossibility of CCD to an (epistemic) multi-person decision-theoretic principle. This might offer a disciplined departure from classical behaviour and appears to be an open direction.

A preference basis for a decision theory with signed probabilities (perhaps, building on Perea [22] or Ke & Zhao [23]) would be of interest in its own right and might also lead to a preference basis for certainty in a non-classical environment. There is a preference basis for certainty in classical decision theory. An agent is certain of an event E if and only if the complementary event $E^{c}$ is Savage-null; that is, if all acts conditional on $E^{c}$ are deemed indifferent. An open question is what would be the analogous definition in the signed case. (We are grateful to Miklós Pintér for raising this question.)

A different non-classical examination of the Agreement Theorem is undertaken by Khrennikov & Basieva [30] and Khrennikov [31]. They consider quantum-like observers of a quantum system who employ either the knowledge or certainty modality. Their approach does not deliver an Agreement Theorem even for quantum systems.

Summing up, our theorem 4.2 establish a new kind of non-classical strangeness in the form of the possibility of CCD. At the same time, we also prove that common knowledge of disagreement and CCD in communication-enabled structures remain impossible (theorems 4.1, 5.4 and 5.6). We believe these results open the door to further investigation of epistemics in non-classical worlds.

## Data Availability

This article has no additional data.
